# Immobilization of Fe-Doped Ni_2_P Particles Within Biomass Agarose-Derived Porous N,P-Carbon Nanosheets for Efficient Bifunctional Oxygen Electrocatalysis

**DOI:** 10.3389/fchem.2019.00523

**Published:** 2019-08-06

**Authors:** Yifan Xiao, Sihui Deng, Meng Li, Qixing Zhou, Libang Xu, Huaifang Zhang, Dongmei Sun, Yawen Tang

**Affiliations:** ^1^Jiangsu Key Laboratory of New Power Batteries, Jiangsu Collaborative Innovation Center of Biomedical Functional Materials, School of Chemistry and Materials Science, Nanjing Normal University, Nanjing, China; ^2^College of Overseas Education, Nanjing Tech University, Nanjing, China

**Keywords:** Fe-Ni_2_P, biomass agarose, N,P-carbon nanosheets, oxygen reduction reaction, oxygen evolution reaction

## Abstract

A feasible and green sol-gel method is proposed to fabricate well-distributed nano-particulate Fe-Ni_2_P incorporated in N, P-codoped porous carbon nanosheets (Fe-Ni_2_P@N,P-CNSs) using biomass agarose as a carbon source, and ethylenediamine tetra (methylenephosphonic acid) (EDTMPA) as both the N and P source. The doped Fe in Ni_2_P is essential for a substantial increase in intrinsic catalytic activity, while the combined N,P-containing porous carbon matrix with a better degree of graphitization endows the prepared Fe-Ni_2_P@N,P-CNSs catalyst with a high specific surface area and improved electrical conductivity. Benefiting from the specific chemical composition and designed active site structure, the as-synthesized Fe-Ni_2_P@N,P-CNSs manifests a satisfying catalytic performance toward both oxygen reduction reaction (ORR) and oxygen evolution reaction (OER) in an alkaline solution, with low overpotential, small Tafel slope and long-term durability, relative to the counterparts (Fe-free Ni_12_P_5_/Ni_2_P_2_O_7_@N,P-CNSs and CNSs) with single components and even comparable to Pt/C and RuO_2_ catalysts. The present work broadens the exploration of efficient bifunctional oxygen electrocatalysts using earth abundant biomass as carbon sources based on non-noble metals for low cost renewable energy conversion/storage.

## Introduction

With the concern of an ever-increasing energy demand as well as serious and intensifying environmental issues, exploring alternative renewable energy sources or energy conversion/storage technologies, instead of fossil fuels, has become more and more pressing to ensure future sustainable development (Zhao et al., 2013; Wang et al., 2015; Chen et al., 2016; Cano et al., 2018). Some examples of these are rechargeable metal-air batteries, water electrolysis and fuel cells, in which electrocatalytic oxygen reduction reaction (ORR) and oxygen evolution reaction (OER) play pivotal and cornerstone roles (Park et al., 2016; Li et al., 2017a; Zheng et al., 2017; Fu et al., 2018a; Pan et al., 2018; Qin et al., 2018). However, both reactions have intrinsically sluggish kinetics with thermodynamic barriers and high overpotentials, originating from their complicated multielectron transfer pathways (Cheng et al., 2017; Jiang et al., 2018; Li et al., 2018a; Fu and Lee, 2019; Fu et al., 2019). Although noble metal-based catalysts have been generally acknowledged as the benchmark for either ORR or OER, such as commercial Pt/C for ORR or RuO_2_/IrO_2_ for OER, their scarcity, rocketing cost and electrochemical instability inevitably limit their large-scale practical implementation (Cheng et al., 2017; Liu et al., 2017a; Wang et al., 2017a, 2018a). Moreover, noble metals can generally not work as efficiently as Janus particles to satisfy the demand for both reactions. Therefore, searching for cost-effective alternatives to substitute noble metals as bifunctional electrocatalysts is extremely significant, yet remains a grand challenge.

Recently, transition metal Ni-based alloys (Fu et al., 2016a,b; Ma et al., 2016; Fu et al., 2018b), hydroxides (Wang et al., 2017b, 2018b; Song et al., 2019), chalcogenides (An et al., 2017; Yin et al., 2017a; Hu et al., 2019a,b; Sumboja et al., 2019), oxides (He et al., 2016; Yin et al., 2017b), nitrides (Wang et al., 2016a; Xie and Xie, 2016; Cui et al., 2017; Fu et al., 2017), as well as phosphides (Wang et al., 2016b, 2017c; Zhang et al., 2016), have sprung up and intensively exploited for ORR/OER, due to their abundance in the earth, tunable surface chemistry and superior electrochemical catalytic activity and stability. Among them, metalloid Ni-based phosphides (Ni_x_P_y_, x,y = 1,2), rich in active hydride acceptor sites with a moderate interaction between phosphorous and reaction intermediate and especially its outstanding practical activity, is an ideal model for in-depth reaction mechanism analysis (Yu et al., 2016). For instance, as demonstrated, the active surface phosphates evolution during OER, positively regulates water oxidation through a proton-coupled electron transfer process (Liu et al., 2017b). A powerful knob to further enhance the catalytic activity of Ni_x_P_y_ is achieved by introducing a second transition metal which effectively modifies surface electronic states (Li and Zhao, 2016; Liu et al., 2017c; Wang et al., 2017d). As a typical example, Mu's group fabricated Fe-doped Ni_2_P nanosheets arrays supported on nickel foam (Ni_1.85_Fe_0.15_P NSAs/NF) via a facile hydrothermal method combined with phosphorization (Wang et al., 2017e), which exhibit advanced OER and hydrogen evolution reaction (HER) performance in water splitting. Sun et al. (2017) demonstrated that the (Ni_0.33_Fe_0.67_)_2_P could work as a bifunctional electrocatalyst with excellent overall water-splitting performance (Li et al., 2017b). Despite the significant progress that has been achieved for Ni_2_P catalysts, most of the works focus on the HER and OER, and rarely on ORR (Sun et al., 2017; Wang et al., 2018c; Yan et al., 2019). On the other hand, the electrocatalytic behavior of Ni_2_P remains unsatisfying to some degree when compared with noble metal-based catalysts. Therefore, it is highly desirable to build a more efficient and robust hybrid architecture for advanced bifunctional oxygen electrocatalytic properties.

In addition, as manifested, heteroatom (e.g., N, P, B, and S)-doped carbon materials hold a relatively high increase in its conductivity owing to the effective tailoring of the electronic state of adjacent carbon atoms and the induced excess defects (Tong et al., 2017; Li et al., 2018b; Fang et al., 2019). Serving as a significant class of raw carbon sources, biomass materials (i.e., chitosan, agarose, sodium alginate, etc.) possess many unique merits, including being widely accessible, rather inexpensive, and environmentally friendly and sustainable, and readiness as a carbon source to fabricate porous materials over simple post-processing (Hao et al., 2017; Li et al., 2018b; Kaur et al., 2019). Hybridizing active Ni_2_P with heteroatoms-doped carbon materials from biomass is therefore a wise strategy to take full advantage of both compositions with synergistic effect. To the best of our knowledge, simple simultaneous incorporation of the selected heteroatoms along with metal precursors into a cost-effective porous agarose gel to generate active Fe-Ni_2_P alloy within N,P-codoped carbon composite catalyst, has rarely been developed.

Herein, we design and exploit the Fe-Ni_2_P particles supported on N,P-codoped porous carbon nanosheets (Fe-Ni_2_P@N,P-CNSs) based on the green biomass of the agarose over sol-gel strategy. Such a facile and easy-to-operate procedure is highly desirable to effectively immobilize active species and reasonably introduce heteroatoms of N and P to the material. Electrochemical studies demonstrate that the synthesized Fe-Ni_2_P@N,P-CNSs deliver desirable bifunctional activities for either ORR or OER, comparable to commercial Pt/C or RuO_2_, respectively. The outstanding electrocatalytic performance toward oxygen electrocatalysis may contribute to the coupling effect between active Fe-Ni_2_P and N,P-codoped porous carbon nanosheets, and the fabricated strategy here is more appealing for scalable preparation of other metal compounds/carbon complex nanomaterials on account of feasibility and versatility.

## Results and Discussion

**Figure 1:**
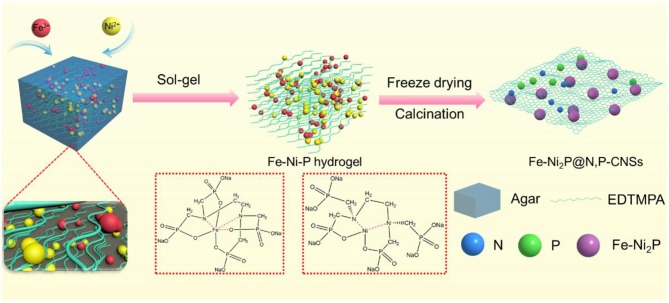
Schematic illustration of the preparation procedure of Fe-Ni_2_P@N,P-CNSs catalyst.

The overall synthetic procedure for the Fe-Ni_2_P@N,P-CNSs was presented in Figure 1. Initially, the precursor solution was first prepared by dissolving iron nitrate, nickel nitrate and EDTMPA together in water. Here the EDTMPA not only effectively interacts with the iron and nickel cations, as demonstrated by UV-visible spectroscopy (Figure S1), but also serves as the dopant precursor of N and P. Then the mixture was transferred to a hot viscous agarose solution at 95°C. The hydroxyl groups in agarose tends to self-assemble and ultimately form a cross-linked hydrogel with metal and N,P precursors evenly embedded inside, when the temperature is dropped to a certain degree as agarose is a temperature sensitive polymer (Zhou et al., 2019). During the fabrication process, the hydrogel agarose not only serves as a smart linker to strongly coordinate with metal ions through abundant oxygen-containing functional groups in the molecular chains, but also as a rational media to effectively circumvent the agglomeration/detachment problem of Fe-Ni_2_P particles during carbothermal reduction. Noticeably, the synthetic approach we proposed here enables lowering the product particle size because of the confinement effect of concrete agarose gel (Zhang et al., 2017; Zhou et al., 2019). After freeze-drying and thermal treatment in an inert atmosphere, the product of Fe-Ni_2_P@N,P-CNSs was finally obtained. The strategy outlined here is flexible, easy to operate and apply in large-scale fabrication.

**Figure 2:**
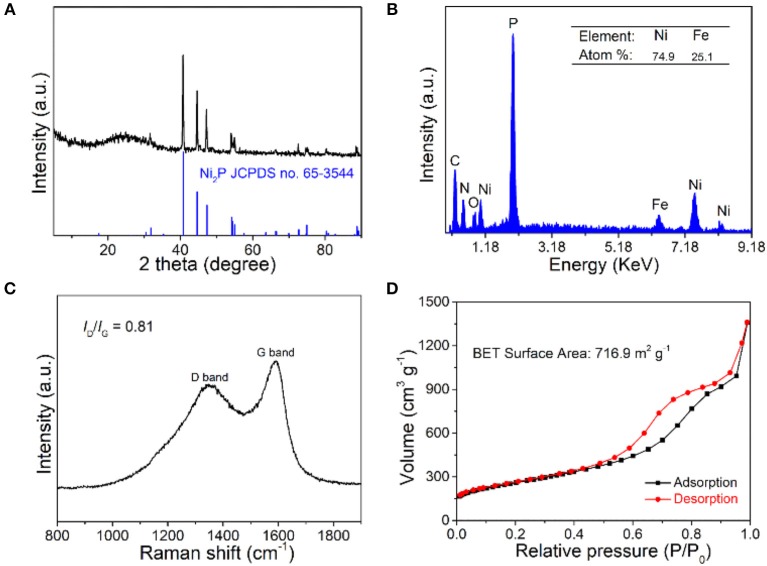
**(A)** XRD pattern, **(B)** EDX spectrum, **(C)** Raman spectra, and **(D)** N_2_ adsorption-desorption isotherm of Fe-Ni_2_P@N,P-CNSs.

The components of Fe-Ni_2_P@N,P-CNSs were first characterized by XRD analysis. As shown in Figure 2A clearly, all the diffraction peaks can be indexed to the Ni_2_P phase (JCPDS no. 65-3544), while no supererogatory diffraction peaks of the Fe phases can be detected, indicating that Fe may be introduced into the lattice of Ni_2_P. The peak at around 26° is attributed to the graphitic carbon (002) crystal plane. Notably, the sample exists in the form of Ni_12_P_5_/Ni_2_P_2_O_7_ when Fe was not involved in the preparation process (Figure S2). The EDX spectrum (Figure 2B) depicts the atom ratio of Ni and Fe as 74.9:25.1 in the product, which is in line with the initial feed ratio of 3:1. To characterize the graphitization degree of Fe-Ni_2_P@N,P-CNSs, Raman spectra were measured as displayed in Figure 2C. The I_D_/I_G_ ratio of Fe-Ni_2_P@N,P-CNSs was calculated to be 0.81, indicating a high graphitization degree of Fe-Ni_2_P@N,P-CNSs, which is beneficial to electron transport when undergoing an electrocatalytic reaction (Fu et al., 2018c; Li et al., 2018c). In order to determine the mass content of Fe-Ni_2_P particles in Fe-Ni_2_P@N,P-CNSs, a TGA measurement was conducted in air atmosphere, as seen in Figure S3. According to the final content of NiO and Fe_3_O_4_, the weight percentage of Fe-Ni_2_P in the complex catalyst was identified as *ca*. 53.84%. To further verify the porous feature, a nitrogen adsorption and desorption experiment was performed (Figure 2D). A significant hysteresis loop appears in the relative pressure (*p*/*p*_0_) range of 0.5 to 1.0, which is consistent with the type IV isotherms. The BET (Brunauer-Emmett-Teller) specific surface area of Fe-Ni_2_P@N,P-CNSs was measured to be about 716.9 m^2^ g^−1^. Note that the large specific area is essential to afford additional active sites with more exposure to electrolytes, so as to deliver enhanced catalytic activity of the catalysts (Fu et al., 2016c).

**Figure 3:**
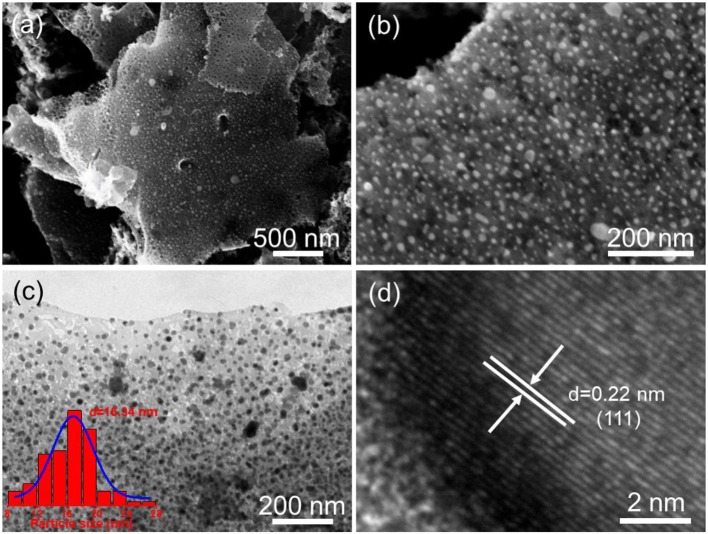
Morphological and structural characterization of the Fe-Ni_2_P@N,P-CNSs: **(a,b)** Typical SEM images, **(c)** TEM image (inset: the corresponding particle-size distribution histogram), and **(d)** HRTEM image.

The morphology and microstructure of the as-obtained Fe-Ni_2_P@N,P-CNSs were initially characterized by scanning electron microscopy (SEM) and transmission electron microscopy (TEM). As observed from the SEM images (Figures 3a,b), the carbon matrix derived from biomass agarose cross-linked well with the Fe-Ni_2_P particles, which suggests that the biomass agarose can effectively capture the Ni^2+^/Fe^3+^ cations in the precursor. The opened porous channel in the carbon matrix stems from the freeze-drying process, which inherits the 3D structural characteristic of hybrid hydrogel intermediates. Such architecture supports more exposed electrocatalytic active sites and provides fast electrolyte penetration, therefore boosting the electrocatalytic activity and durability (Fu et al., 2018d). TEM image (Figure 3c) further reveals a predominant 2D sheet-like carbon structure encapsulated with high density Fe-Ni_2_P particles, and the average size distribution of Fe-Ni_2_P particles is established to be around 16.34 nm. The HRTEM image (Figure 3d) reveals an individual Fe-Ni_2_P particle, and the well-resolved interplanar distances are measured to be 0.22 nm, which is slightly smaller than that of the {111} plane of Ni_2_P (0.221 nm). These results indicate that the element of Fe is successfully incorporated into the lattice of Ni_2_P via partial substitution and eventually forms Fe-Ni_2_P. The energy dispersive X-ray (EDX) elemental mapping analyses (Figure S4) demonstrate that all the elements, including Ni, Fe, O, N, P, C are homogenously distributed throughout the entire Fe-Ni_2_P@N,P-CNSs nanocomposite. For comparison, Ni_12_P_5_/Ni_2_P_2_O_7_@N,P-CNSs and pure CNSs were also synthesized through similar steps to Fe-Ni_2_P@N,P-CNSs, except without the addition of the Fe nitrates, and EDTMPA and Ni/Fe nitrates, respectively. The TEM images are shown in Figure S5. As observed, the Ni_12_P_5_/Ni_2_P_2_O_7_@N,P-CNSs possess distinct particles within porous carbon sheets, while CNSs only present a smooth surface of the carbon nanosheet, which may highlight that the inclusion of metal nitrate species is facilitated to form well-defined porous carbon sheets due to the catalytic effect.

**Figure 4:**
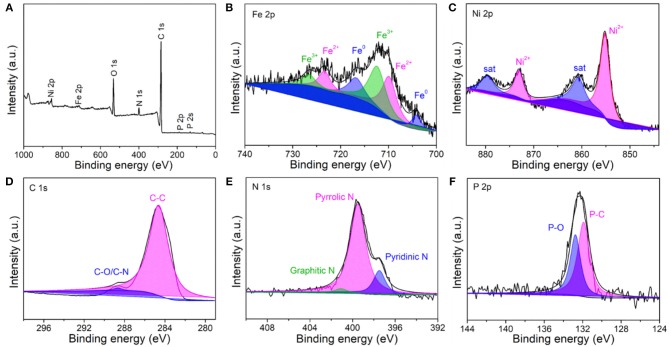
XPS spectra of the Fe-Ni_2_P@N,P-CNSs: **(A)** survey scan spectrum, high-resolution spectra of **(B)** Fe 2p, **(C)** Ni 2p, **(D)** C 1s, **(E)** N 1s, and **(F)** P 2p.

X-ray photoelectron spectroscopy (XPS) measurements were performed to investigate the chemical composition and the electronic structure of the resultant Fe-Ni_2_P@N,P-CNSs. The XPS survey spectrum (Figure 4A) confirms the co-existence of C, N, O, P, Fe and Ni elements in the sample. In high-resolution Fe 2p spectrum (Figure 4B), two predominant peaks centered at 712.58 (Fe 2p_3/2_) and 726.68 eV (Fe 2p_1/2_) are ascribed to Fe^3+^, while the peaks located at 709.98 (Fe 2p_3/2_), 723.38 eV (Fe 2p_1/2_) and 703.98 (Fe 2p_3/2_), 716.68 eV (Fe 2p_1/2_) relate to Fe^2+^ and Fe^0^ species, respectively. The high-resolution Ni 2p spectrum (Figure 4C) exhibits two predominant peaks at 855.10 eV (Ni 2p_3/2_) and 873.12 eV (Ni 2p_1/2_), along with two weak shakeup satellite peaks at 861.0 and 879.6 eV. These four peaks are assigned to Ni^2+^ cations, which may derive from the surface oxidation in air condition. The deconvoluted high-resolution C 1s spectrum of the Fe-Ni_2_P@N,P-CNSs is presented in Figure 4D. The C 1s spectrum shows prominent peaks located at 284.59 and 288.68 eV, associated to the chemical compositions of C-C and C-N/C-O in the carbon matrix. Observed from the high-resolution N 1s spectrum (Figure 4E), three types of N configurations can be classified into pyrrolic N (399.52 eV), pyridinic N (397.47 eV) and graphitic N (401.14 eV), respectively. Additionally, for the high-resolution P 2p spectrum (Figure 4F), it shows that the P-C bond centered at about 131.83 eV and P-O at around 132.79 eV. The existence of N and P is generally believed to be of great value to the electro-catalysis of OER and ORR owing to the electronic modification effect (Hu et al., 2017; Wang et al., 2018d). These results have also confirmed the effective incorporation of N and P into the carbon matrix and the successful formation of the Fe-Ni_2_P nanophase.

**Figure 5:**
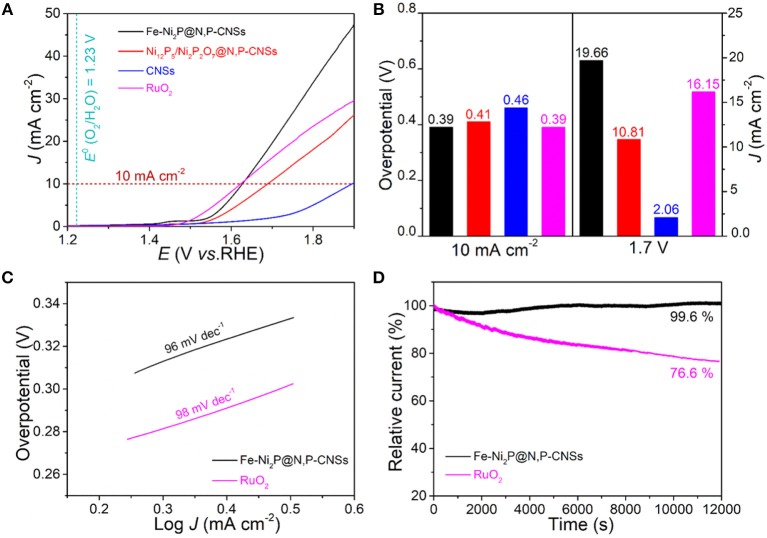
Comparison of the electrochemical OER performance of Fe-Ni_2_P@N,P-CNSs, Ni_12_P_5_/Ni_2_P_2_O_7_@N,P-CNSs, CNSs and commercial RuO_2_ catalysts: **(A)** OER polarization curves, **(B)** overpotentials at 10 mA cm^−2^ and current densities achieved at 1.7 V, **(C)** corresponding Tafel plots, and **(D)** chronopotentiometry curves of the Fe-Ni_2_P@N,P-CNSs and RuO_2_ catalyst at 1.6 V in O_2_-saturated 0.1 M KOH.

As inspired by the advantages of the structure and compositions of the as-fabricated catalyst, the resultant Fe-Ni_2_P@N,P-CNSs may hold great promise as an enhanced electrocatalyst for bifunctional oxygen electrocatalysis. The electrocatalytic properties of Fe-Ni_2_P@N,P-CNSs electrode toward OER and ORR were performed in a 0.1 M KOH solution. For comparison, CNSs, Fe-free Ni_12_P_5_/Ni_2_P_2_O_7_@N,P-CNSs, commercial RuO_2_ (0.56 mg cm^−2^) and Pt/C catalyst (0.56 mg cm^−2^) were also evaluated under the identical test conditions. For the OER process, Figure 5A depicts the typical linear sweep voltammetry (LSV) curves of the studied catalysts with a scan rate of 5 mV s^−1^. Apparently, the Fe-Ni_2_P@N,P-CNSs catalyst exhibits the highest OER activity than any other sample. The overpotential to achieve the current density of 10 mA cm^−2^ for Fe-Ni_2_P@N,P-CNSs is as low as 0.39 V, which is comparable with benchmark RuO_2_ (0.39 V) and much lower than that of Ni_12_P_5_/Ni_2_P_2_O_7_@N,P-CNSs (0.41 V), and CNSs (0.46 V), respectively. The Fe-Ni_2_P@N,P-CNSs also exhibits a higher current density (19.66 mA cm^−2^) than RuO_2_ (16.15 mA cm^−2^), Ni_12_P_5_/Ni_2_P_2_O_7_@N,P-CNSs (10.81 mA cm^−2^) and CNSs (2.06 mA cm^−2^) under the potential of 1.7 V (Figure 5B), signifying the superior activity of the Fe-Ni_2_P@N,P-CNSs in the electrochemical implementation. The Tafel slope is employed as an intrinsic parameter to evaluate the catalytic reaction kinetic of the synthesized hybrids. As depicted in Figure 5C, the Tafel slope of Fe-Ni_2_P@N,P-CNSs is calculated to be 96 mV dec^−1^, slightly smaller than that of commercial RuO_2_ (98 mV dec^−1^), suggesting favorable kinetics for the high-efficiency of mass and electron transfer behavior. Given the high current density and minor Tafel slope of Fe-Ni_2_P@N,P-CNSs, the catalyst exemplifies excellent OER performance. The outstanding electrocatalyst properties may originate from the synergistic effect of Fe-Ni_2_P and N,P-codoped carbon sheets. To the best of our knowledge, the OER performance of Fe-Ni_2_P@N,P-CNSs is comparable or even superior over many other non-noble OER catalysts that have recently been reported (Table S1). To gain further insights into the active sites available in our fabricated Fe-Ni_2_P@N,P-CNSs, relative to commercial RuO_2_, the different scan rates of CV curves within the potential window range of 0.94-1.04 V in non-Faradaic region were recorded as shown in Figure S6, since the electrochemical active surface area (ECSA) is believed to be positively proportional to electrochemical double-layer capacitance (*C*_dl_). The *C*_dl_ value of Fe-Ni_2_P@N,P-CNSs (6.6 mF cm^−2^) is measured slightly higher than that of RuO_2_ (6.2 mF cm^−2^), suggesting that Fe-Ni_2_P@N,P-CNSs possess more utilizable active sites. The electrochemical impedance spectra (EIS) of the three types of electrodes are shown in Figure S7. It is known that a semicircle in the high-frequency range of the Nyquist plot reflects the charge-transfer resistance R_ct_, and the lower the value of R_ct_ is, the faster the kinetic rate is. The Fe-Ni_2_P@N,P-CNSs electrode exhibits the lowest value of R_ct_, demonstrating the superior electrocatalytic kinetics and more efficient activity than Ni_12_P_5_/Ni_2_P_2_O_7_@N,P-CNSs and CNSs under the OER. The long-term catalytic durability is also another critical parameter for practical applications, which is assessed by chronoamperometric measurements (*i-t*). As shown in Figure 5D, after *i-t* test at 1.6 V for 12,000 s, the current density retention of Fe-Ni_2_P@N,P-CNSs is 99.6%, which is much better than that of RuO_2_ (76.6%) under the identical potential. The robust stability of Fe-Ni_2_P@N,P-CNSs may benefit from its strong intimate contact with the carbon matrix. The carbon outer layer also protects the active particles from agglomeration or detachment during the measurement process, thus contributing to maintaining the electrode structure integrity.

**Figure 6:**
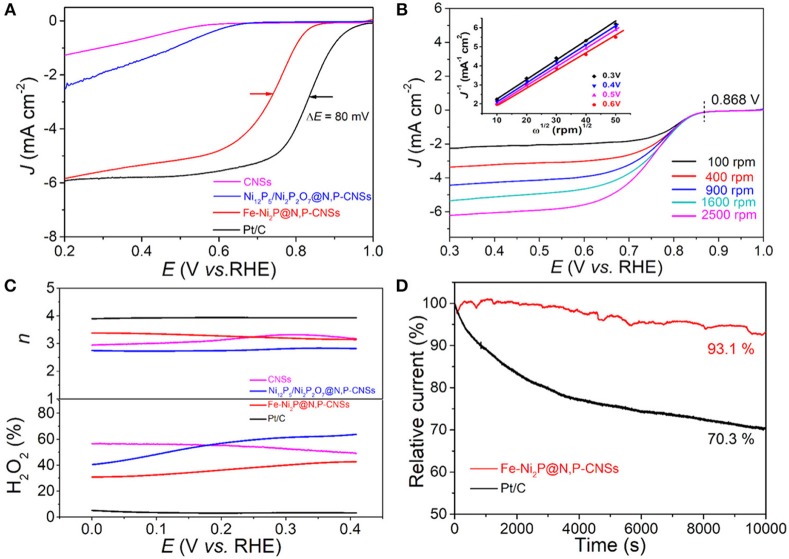
Electrocatalytic ORR performance of studied catalysts: **(A)** Linear scan voltammogram (LSV) curves for Fe-Ni_2_P@N,P-CNSs, Ni_12_P_5_/Ni_2_P_2_O_7_@N,P-CNSs, CNSs and commercial Pt/C catalyst at 1,600 rpm in O_2_-saturated 0.1 M KOH solution at 5 mV s^−1^. **(B)** LSV curves of Fe-Ni_2_P@N,P-CNSs in O_2_-saturated 0.1 M KOH at various rotating speeds (100–2,500 rpm). Inset is the corresponding Koutecky–Levich plots of Fe-Ni_2_P@N,P-CNSs at different potentials. **(C)** Percentage of the peroxide with respect to the total oxygen reduction products and corresponding electron transfer number n at different potentials. **(D)** Chronoamperometric responses of the Fe-Ni_2_P@N,P-CNSs and Pt/C at 0.6 V vs. RHE in an O_2_-saturated 0.1 M KOH solution.

To gain insight into the ORR properties of the as-obtained catalysts, we first evaluated the electrochemical behavior of Fe-Ni_2_P@N,P-CNSs, Ni_12_P_5_/Ni_2_P_2_O_7_@N,P-CNSs and CNSs by LSV technique with a 1,600 rpm rotation rate at 5 mV s^−1^ in an O_2_-saturated 0.1 M KOH solution (Figure 6A). The onset potential (*E*_0_, the potential at −10 μA cm^−2^) and half-wave potential (*E*_1/2_) are two significant parameters to verify the ORR activities. The Fe-Ni_2_P@N,P-CNSs show a more positive onset potential (0.978 V vs. RHE), approaching that of the Pt/C catalyst (1.01 V vs. RHE). In terms of *E*_1/2_, the *E*_1/2_ of Fe-Ni_2_P@N,P-CNSs is at 0.75 V, which is 80 mV more negative than that of commercial Pt/C (0.83 V vs. RHE). Moreover, a relatively high diffusion-limiting current density of the Fe-Ni_2_P@N,P-CNSs (5.82 mA cm^−2^ at 0.2 V) is comparable to that of Pt/C (5.83 mA cm^−2^). The ORR activity of Fe-Ni_2_P@N,P-CNSs is also comparable to other recently reported transition metal-based ORR electrocatalysts (Table S2). The remarkable ORR activity of Fe-Ni_2_P@N,P-CNSs may originate from the synergistic effect between Fe-Ni_2_P particles and N,P-codoped carbon sheets. To quantitatively understand the ORR behavior of the resultant catalyst, detailed LSV curves with different rotation rates ranging from 100 to 2,500 rpm of the Fe-Ni_2_P@N,P-CNSs are shown in Figure 6B. It is clearly observed that the diffusion-limiting polarization curves reach a well-defined plateau, and the corresponding Koutecky–Levich plots (inset in Figure 6B) is shown to analyze the ORR kinetics. The electron transfer number (n) of the ORR can be calculated from the slope of the fitted lines. Based on the average values calculated at different potentials, n was estimated to be 3.2–3.3 in the potential window range of 0.3–0.6 V, demonstrating that the Fe-Ni_2_P@N,P-CNSs favor a mix of desirable 4e^−^ and 2e^−^ reduction reaction pathways. RRDE measurements were also performed to monitor the ORR pathway to the Fe-Ni_2_P@N,P-CNSs. The peroxide (HO${}_{2}^{-}$) yield percentage and the electron transfer number n were established from the corresponding disk and ring currents (Figure 6C). The ORR on the Fe-Ni_2_P@N,P-CNSs yielded about 30–42% HO${}_{2}^{-}$ during the potential range of 0-0.4 V, and the *n* was calculated to be 3.4 to 3.2, in agreement with results obtained from the Koutecky–Levich plots. Stability is another vital criterion to assess practical ability. Therefore, the Fe-Ni_2_P@N,P-CNSs and Pt/C were also examined with chronoamperometric measurements at 0.6 V vs. RHE for 10,000 s in O_2_-saturated 0.1 M KOH solution (Figure 6D). After 10,000 s of continuous operation, the ORR current density of the Pt/C decreased by 29.7%, while a decrease of only 6.9% in the current density was detected for the Fe-Ni_2_P@N,P-CNSs. On the basis of the above statements, we reasonably expect that the Fe-Ni_2_P@N,P-CNSs can be employed as outstanding bifunctional electrocatalyst for both OER and ORR.

The outstanding electrocatalytic performance of Fe-Ni_2_P@N,P-CNSs toward OER and ORR can be ascribed to the structural and composition features as follows: (i) the strong coupling effect between the well-dispersed Fe-Ni_2_P particles and conductive porous carbon nanosheets, which is facile for the exposure of more active sites (Hu et al., 2016; Lei et al., 2018); (ii) the introduction of N and P in the carbon matrix, by which the electronic structure of the applied carbon support is effectively modified, creates more available defects with enhanced catalytic activity and durability (Huang et al., 2018; Wei et al., 2018); (iii) the large specific surface area of Fe-Ni_2_P@N,P-CNSs, which provides numerous accessible catalytic sites, and endows available infiltration to the electrolyte for effective O_2_ bubble evolution and a reductive reaction over the electrode surface (Fu et al., 2018e; Zhou et al., 2018). By taking advantage of these exceptional structural merits and the synergistic effect between the multi-components, the as-prepared Fe-Ni_2_P@N,P-CNSs display impressive catalytic activity and stability toward both OER and ORR.

## Conclusion

In summary, we have demonstrated a simple and scalable approach to fabricate Fe-Ni_2_P@N,P-CNS electrocatalysts by taking EDTMPA-coordinated metal and biomass agarose as precursors, combined with and followed by a thermal-annealing treatment. We found that the introduction of Fe atoms into the Ni_2_P can effectively afford more catalytic active sites, which play a crucial role in enhanced electrocatalytic properties. Thanks to the synergic interactions of multi-components and the merits of porosity, conductivity and structural defects, the Fe-Ni_2_P@N,P-CNSs exhibit excellent electrocatalytic performance as a bifunctional electrocatalyst for both ORR and OER in terms of determined activity and stability in alkaline media. The present work opens a new avenue to rationally construct effective bifunctional oxygen electrocatalysts in the domain of energy storage and conversion applications.

## Experimental Section

### Reagents and Chemicals

Agarose powder was purchased from Beijing Solarbio Science and Technology Co., Ltd. Nickel nitrate hexahydrate (Ni(NO_3_)_2_·6H_2_O) and Iron nitrate non-ahydrate (Fe(NO_3_)_3_·9H_2_O) were supplied by Sinopharm Chemical Reagent Beijing Co., Ltd. Ethylenediamine tetra (methylenephosphonic acid) (EDTMPA) was obtained from Aladdin-reagent. Ammonium hydroxide (NH_3_·H_2_O) and Nitric acid (HNO_3_) were received from the Sinopharm Chemical Reagent. Other reagents were of analytical reagent grade and used as received without further purification.

### Synthesis of Fe-Ni_2_P@N,P-CNSs Sample

In a typical reaction, 0.1744 g of Ni (NO_3_)_2_·6H_2_O and 0.0808 g Fe (NO_3_)_3_·9H_2_O were first dissolved in 2 ml distilled water to prepare a uniform mixture solution, then ammonium hydroxide was added to a 5 ml 0.2 M EDTMPA solution to adjust the pH value to 9.0. Afterwards, the Ni/Fe-EDTMPA complex was synthesized by slowly adding the 0.2 M EDTMPA solution dropwise until the color of the solution turned to emerald green. Subsequently, 0.2 g agarose powder was dissolved in 12 ml distilled water and kept stirred at 95°C for 30 min to form a homogeneous sol-gel. The formed hot sol-gel was then transferred to the metal precursor solution, which changed into hydrogel after being cooled to room temperature. The produced hydrogel was freeze-dried, then calcinated at 750°C at the heating rate of 5°C min^−1^ for 6 h under a N_2_ atmosphere. The resultant material was centrifuged with distilled water and ethanol three times, respectively, to remove impurities completely. Finally, the washed sample was dried in a vacuum dryer at 60°C overnight to obtain the Fe-Ni_2_P@N,P-CNSs. For comparison, the samples of Ni_12_P_5_/Ni_2_P_2_O_7_ immobilized in N,P-codoped porous carbon nanosheets (Ni_12_P_5_/Ni_2_P_2_O_7_@N,P-CNSs) and pure porous carbon nanosheets (CNSs) were also synthesized under similar experimental conditions without Fe nitrates, and EDTMPA and Ni/Fe nitrates, respectively.

### Characterization

The morphology was determined with transmission electron microscopy (TEM) by a JEOL JEM-2100F transmission electron microscope and field-emission scanning electron microscopy (FESEM) images were taken on a JEOL JSM7500F. Energy dispersive spectrum (EDS), high-angle annular dark-field scanning transmission electron microscopy (HAADF-STEM) and elemental mapping images were conducted on an FEI Tecnai G2 F20 microscope, which is built as an accessory to the JEOL JEM-2100F. X-ray diffraction (XRD) patterns were recorded on a Model D/max-rC X-ray diffractometer using the Cu Karadiation source (λ = 1.5406 Å) operating at 40 kV and 100 mA. XPS measurements were conducted on a Thermo VG Scientific ESCALAB 250 spectrometer with an Al Kα radiator, and the binding energy was calibrated by means of the C 1s peak energy of 284.6 eV. Ultraviolet and visible spectroscopy (UV-vis) spectra were recorded at room temperature on a Shimadzu TU-1900 spectrophotometer equipped with 1.0 cm quartz cells. Raman spectroscopy was performed using a LabRam HR800 microscopic laser confocal Raman spectrometer. The Brunauer-Emmett-Teller (BET) Surface area and porosity analysis were analyzed using the ASAP2050 system from Micromeritics Instrument, USA. Thermogravimetric analysis (TGA) was carried out under air with a temperature ramp of 10°C·min^−1^ using a Netzsch SSTA 449C thermogravimetric analyzer (Netzsch STA 449C).

### Electrochemical Measurement

All electrochemical tests were performed on a CHI 760D electrochemical analyzer (Shanghai, Chenghua Co., Ltd.) equipped with high-speed rotators from Gamry Instruments. A conventional three-electrode system was employed, the system consisted of a graphite rod as the auxiliary electrode, a saturated calomel reference electrode protected by a Luggin capillary with KCl solution as the reference electrode, and a rotating disk electrode (RDE) or rotating ring-disk electrode (RRDE) as the working electrodes (0.196 cm^2^). Typically, 5 mg of the sample was ultrasonicated in a mixture of 0.90 ml ethanol and 0.10 ml Nafion solution (5 wt %) for 40 min to form a homogeneous ink, then 5 μl catalyst ink was dropped on the surface of a glassy carbon electrode and dried at room temperature. OER, ORR electrocatalytic tests were carried out in an oxygen-saturated 0.1 M KOH solution. The reference potential in this work was converted to the reversible hydrogen electrode (RHE) via the following equation: *E*_RHE_ = *E*_SCE_ + 0.241 V + 0.0591 pH. All the electrochemical measurements were carried out at 30 ± 1°C. The percentage of hydrogen peroxide (%HO${}_{2}^{-}$) and the electron transfer number (*n*) were calculated according to the following equations:

(1) $$\begin{array}{r} \%H{O_{2}}^{-}=\frac{200I_{r}}{NI_{d}+I_{r}} \end{array}$$

(2) $$\begin{array}{r} n=\frac{4NI_{d}}{NI_{d}+I_{r}} \end{array}$$

Where *I*_r_ is the ring current density, *I*_d_ is the disk current density, and N is the collection efficiency for the Pt ring, which was determined to be 0.37.

## Data Availability

All datasets generated for this study are included in the manuscript and/or the Supplementary Files.

## Author Contributions

YT and DS planned all the experiments. YX, SD, ML, QZ, LX, and HZ synthesized the samples and performed the electrochemical measurements. YT and DS wrote the manuscript and all authors have read and approved it.

### Conflict of Interest Statement

The authors declare that the research was conducted in the absence of any commercial or financial relationships that could be construed as a potential conflict of interest.
