# Supplementary material for: Immobilization of Fe-Doped Ni2P Particles Within Biomass Agarose-Derived Porous N,P-Carbon Nanosheets for Efficient Bifunctional Oxygen Electrocatalysis
Source: Front Chem. 2019 Aug 6;7:523. doi: 10.3389/fchem.2019.00523 (PMC6691339; doi:10.3389/fchem.2019.00523)
Supplement: Supplementary file 1 [file Data_Sheet_1.docx]

***Supplementary Material***

**Part I: Figures**


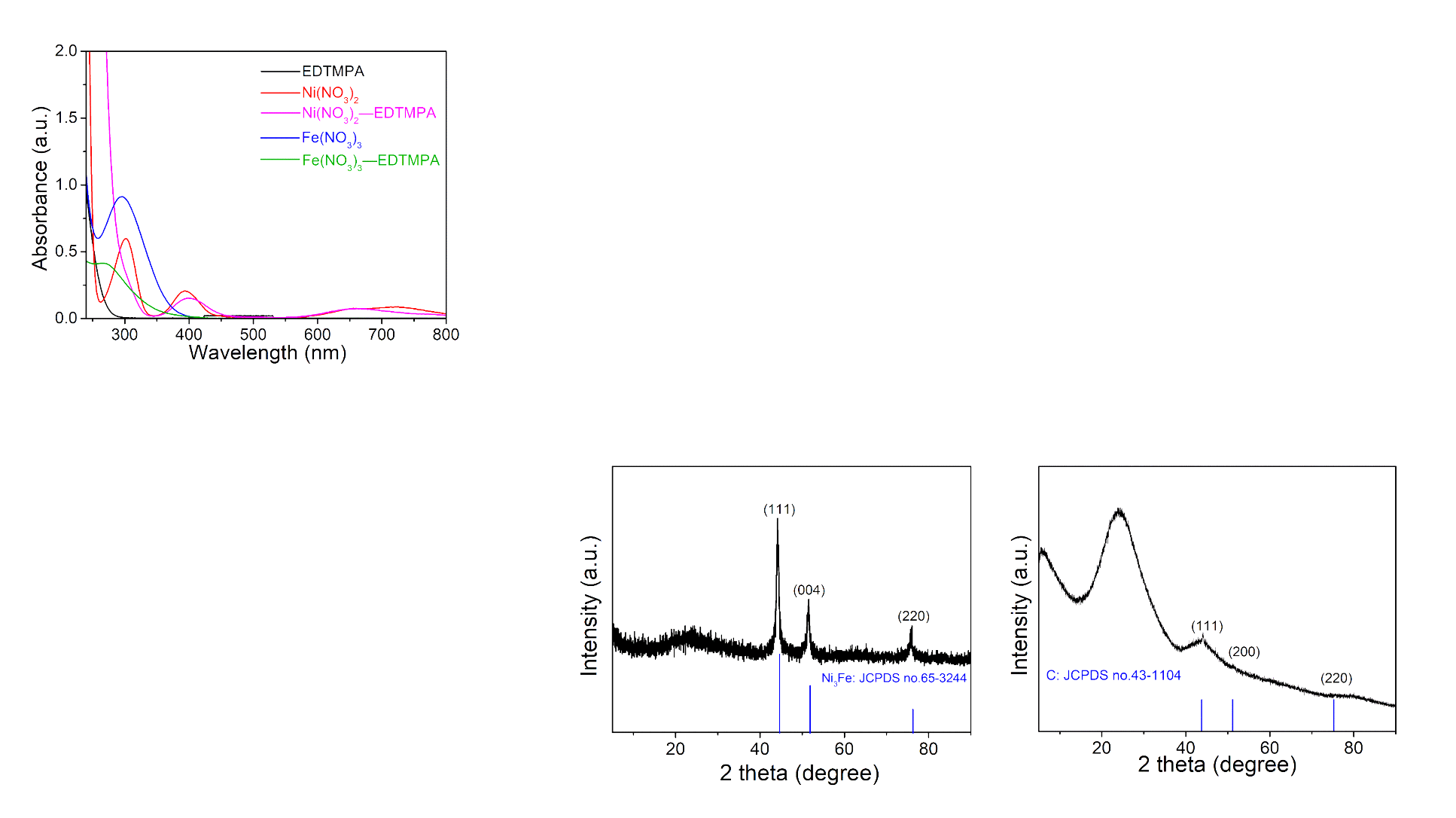


**Figure S1**. UV-vis absorption spectra of different samples.


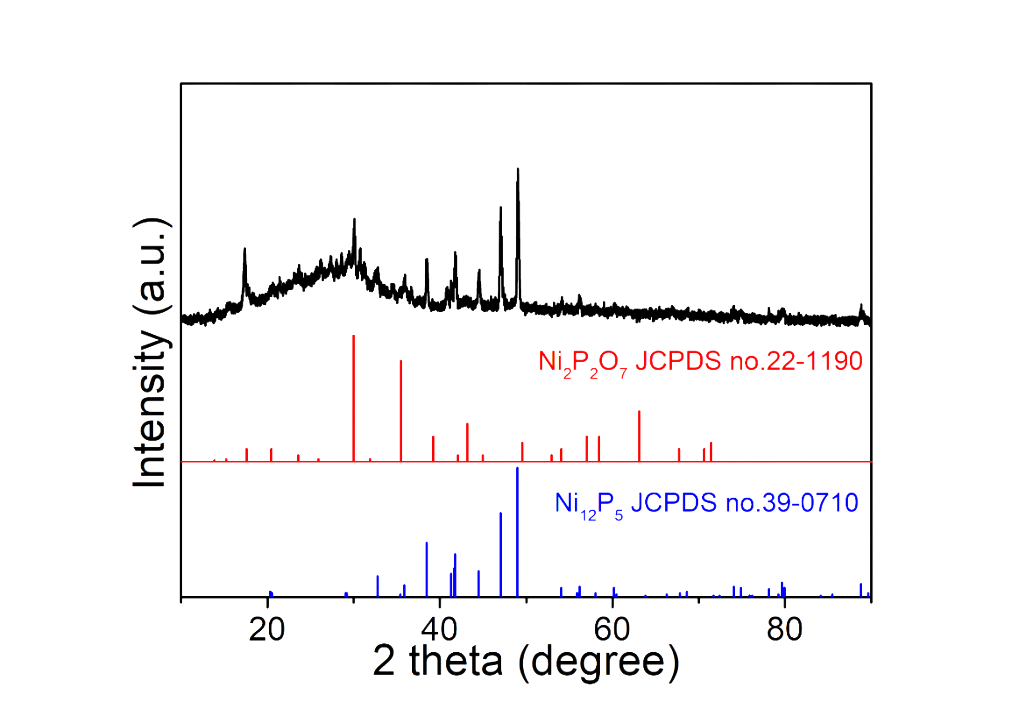


**Figure S2.** XRD patterns of Ni_12_P_5_/Ni_2_P_2_O_7_@N,P-CNSs.


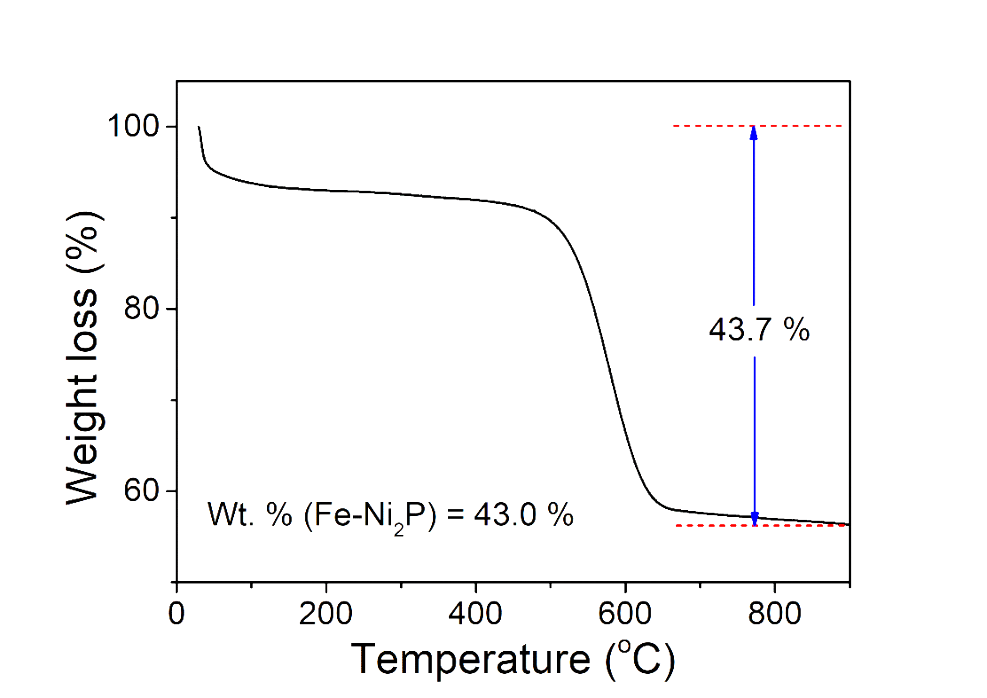


**Figure S3**. TG curve of the obtained Fe-Ni_2_P@N,P-CNSs.


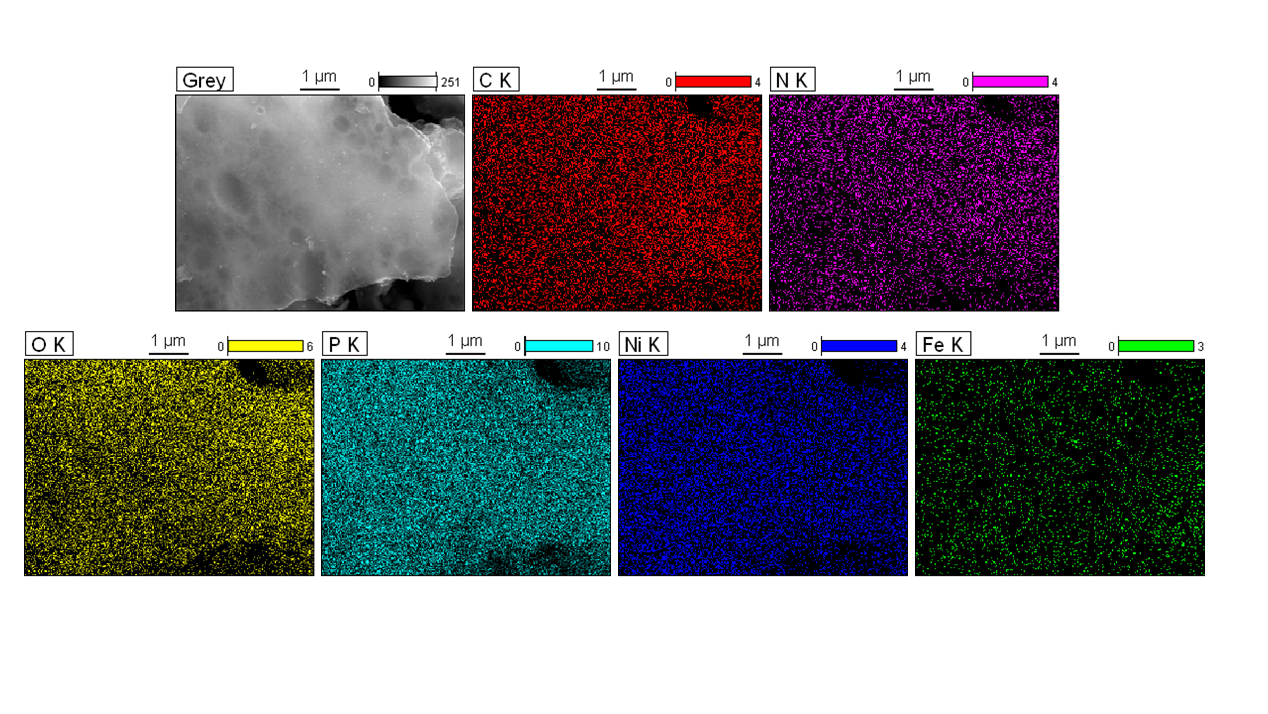


**Figure S4**. HAADF-STEM mapping images of the obtained Fe-Ni_2_P@N,P-CNSs.


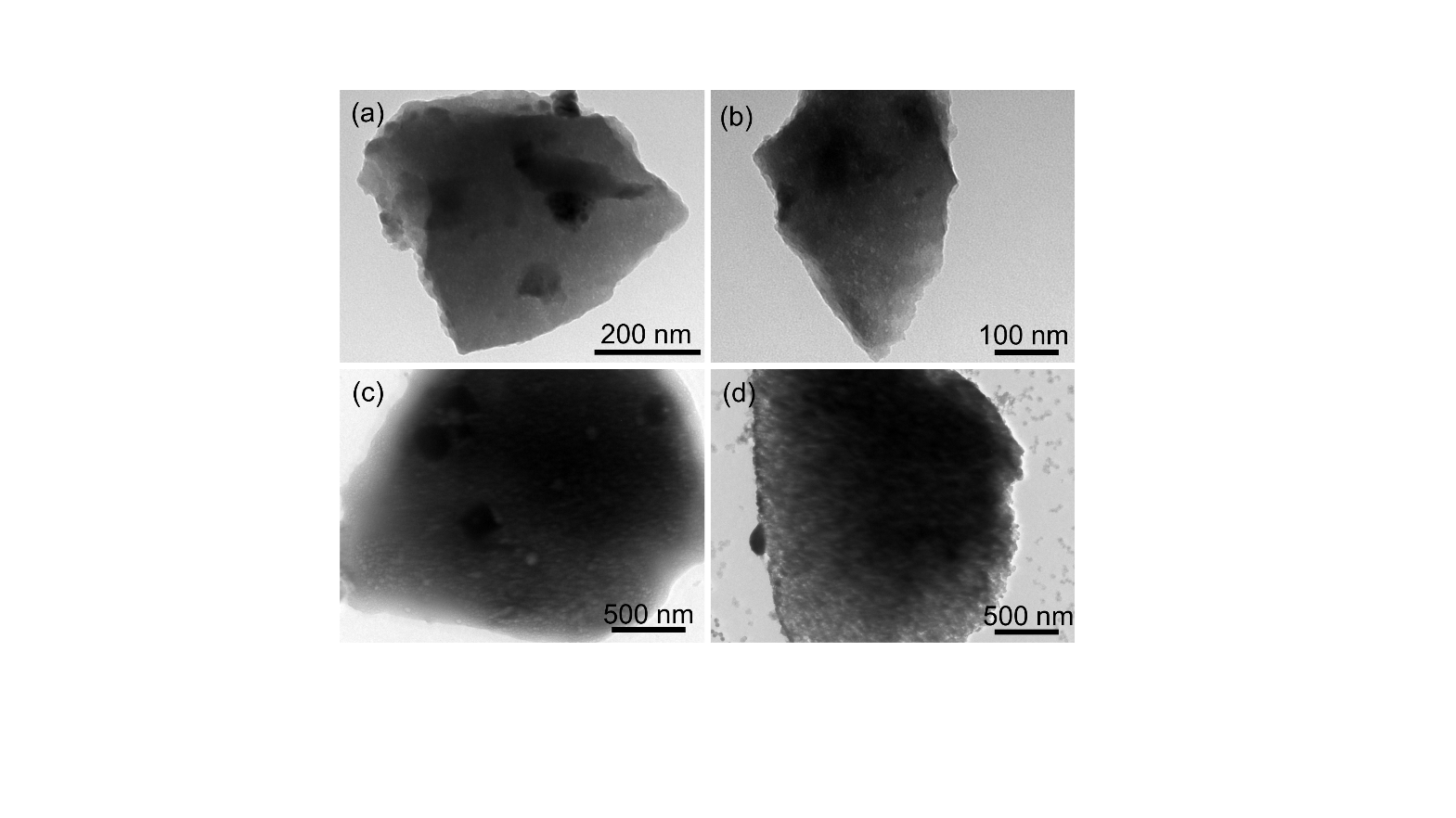


**Figure S5.** TEM images of (a, b) CNSs and (c, d) Ni_12_P_5_/Ni_2_P_2_O_7_@N,P-CNSs.


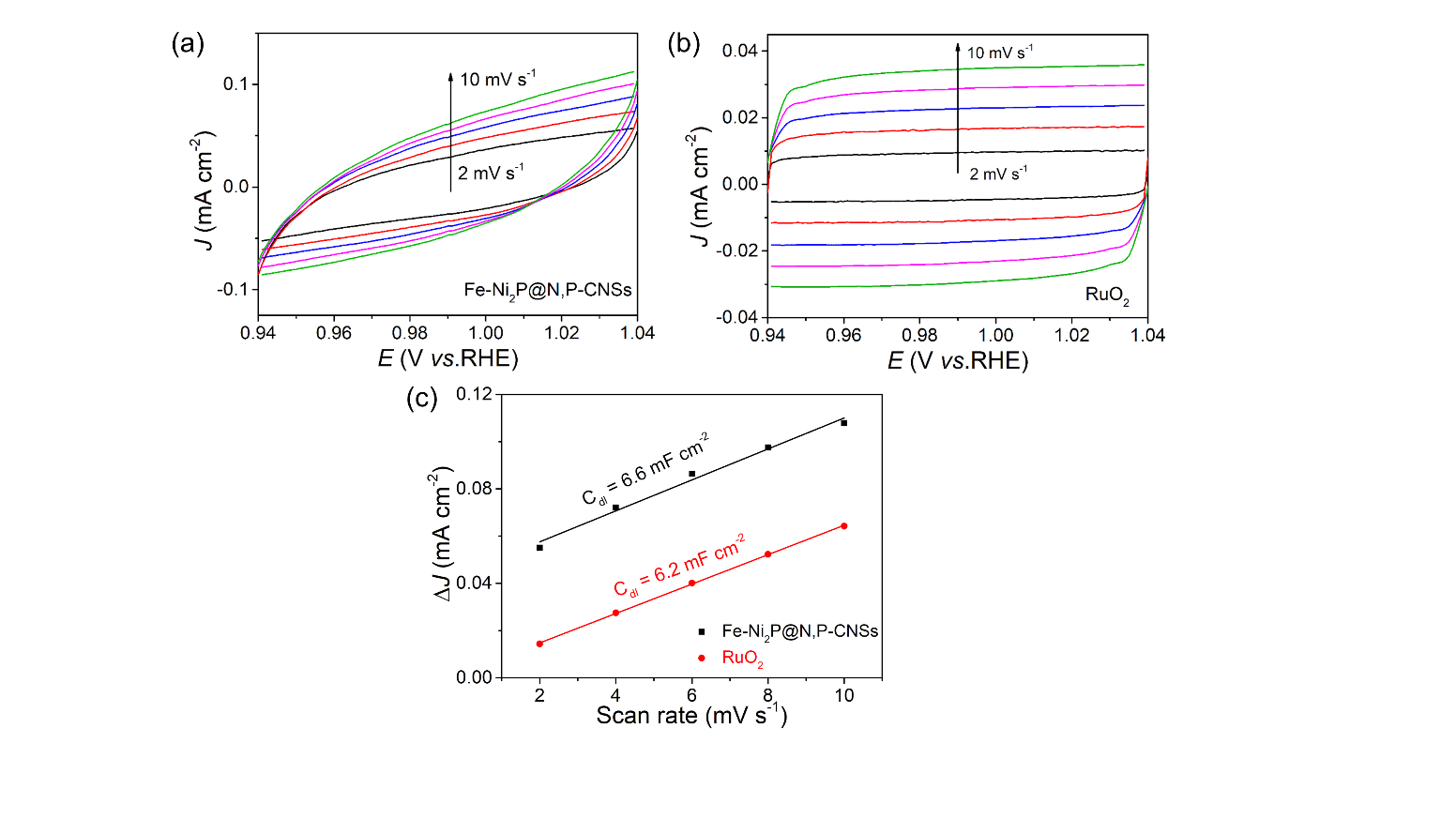


**Figure S6.** Electrochemical surface area (ESCA) tests of Fe-Ni_2_P@N,P-CNSs and commercial RuO_2_ catalysts recorded in N_2_-saturated 0.1 M KOH. (a) CV curves of Fe-Ni_2_P@N,P-CNSs and (b) commercial RuO_2_ catalyst in the non-Faradaic region obtained at different scanning rates. (c) Linear fitting of current density as a function of the scan rate for the different electrodes.


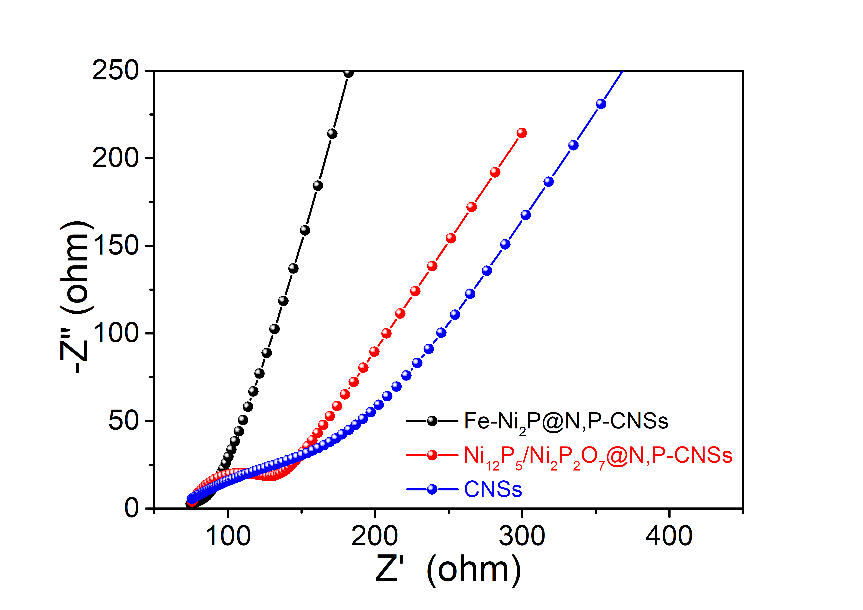


**Figure S7.** EIS spectra (Nyquist plots) of obtained catalysts recorded at 1.6 V in 0.1 M KOH solution.

**Part II. Tables**

**Table S1**. Comparison of the OER activity of the Fe-Ni_2_P@N,P-CNSs with other electrocatalysts previously reported in alkaline medium.

| Catalyst | Mass loading  (μg cm^-2^) | Potential / V  (10 mA cm^-2^) | Tafel slop  (mV dec^-1^) | Electrolyte | Ref |
| --- | --- | --- | --- | --- | --- |
| CuCo_2_O_4_ | 200 | 1.700 | N/A | 1 M KOH | S1 |
| Fe/Fe_3_C@NGL-NCNT | 102 | 1.880 | N/A | 0.1 M KOH | S4 |
| CMO/rGO | 1140 | 1.920 | N/A | 0.1 M KOH | S6 |
| N, S-CN | 200 | 1.680 | 59 | 0.1 M KOH | S8 |
| Co/C/N | 200 | 1.610 | N/A | 0.1 M KOH | S9 |
| Ni/α-MnO_2_-SF | 204 | 1.508 | 107.4 | 0.1 M KOH | S10 |
| Co/MnO@GC-800 | N.A. | 1.417 | 145 | 1 M KOH | S11 |
| Fe-Ni_2_P@N,P-CNSs | 560 | 1.62 | 96 | 0.1 M KOH | This work |

**Table S2.** Comparison of the ORR activity of the Fe-Ni_2_P@N,P-CNSs with other electrocatalysts previously reported.

| Catalyst | Mass loading  (μg cm^-2^) | Onset potential  V (*vs*. RHE) | half-wave potential  V (*vs*. RHE) | Electrolyte | Ref |
| --- | --- | --- | --- | --- | --- |
| CuCo_2_O_4_ | 200 | N/A | 0.800 | 1M KOH | S1 |
| GaSe | N.A | 0.728 | 0.700 | 0.1 M KOH | S2 |
| Co/CoO@Co–N-C-800 | 305 | 0.950 | 0.830 | 0.1 M KOH | S3 |
| Fe/Fe_3_C@NC | 102 | 0.810 | N/A | 0.1 M KOH | S4 |
| CN–CFP | 200 | 0.900 | 0.630 | 0.1 M KOH | S5 |
| CMO/N-rGO | 1140 | 0.900 | 0.800 | 0.1 M KOH | S6 |
| N, S-CN | 200 | 0.950 | 0.800 | 0.1 M KOH | S8 |
| Fe/C/N | 200 | 0.950 | 0.830 | 0.1 M KOH | S9 |
| α-MnO_2_-SF | 204 | 0.870 | 0.780 | 0.1 M KOH | S10 |
| Fe-Ni_2_P  @N,P-C NSs | 560 | 0.978 | 0.722 | 0.1 M KOH | This work |

**References**

[S1] A. Serov, N. I. Andersen, A. J. Roy, I. Matanovic, K. Artyushkova and P. Atanassov, *J. Electrochem. Soc.* 2015, **162**, F449-F454.

[S2] S. M. Tan, C. K. Chua, D. Sedmidubsky, Z. C. Sofer and M. Pumera, *Phys. Chem. Chem. Phys*. 2016, **18**, 1699-1711.

[S3] X. Zhang, R. Liu, Y. Zang, G. Liu, G. Wang, Y. Zhang, H. Zhang and H. Zhao, *Chem Commun.* 2016, **52**, 5946-5949.

[S4] J. S. Li, S. L. Li, Y. J. Tang, M. Han, Z. H. Dai, J. C. Bao and Y. Q. Lan, *Chem Commun.* 2015, **51**, 2710-2713.

[S5] T. Y. Ma, J. Ran, S. Dai, M. Jaroniec and S. Z. Qiao, *Angew. Chem. Int. Ed.* 2015, **54**, 4646-4650.

[S6] M. Prabu, P. Ramakrishnan and S. Shanmugam, *Electrochem. Commun.* 2014, **41**, 59-63.

[S7] J. S. Lee, G. S. Park, H. I. Lee, S. T. Kim, R. Cao, M. Liu and J. Cho, *Nano Lett*. 2011, **11**, 5362-5366.

[S8] K. Qu, Y. Zheng, S. Dai and S. Z. Qiao, *Nano Energy* 2016, **19**, 373-381.

[S9] Y. Zhao, K. Kamiya, K. Hashimoto and S. Nakanishi, *J. Phys. Chem. C*, 2015, **119**, 2583-2588.

[S10] Y. Meng, W. Song, H. Huang, Z. Ren, S. Y. Chen and S. L. Suib, *J. Am. Chem. Soc*. 2014, **136**, 11452-11464.

[S11] J. Xu, H. Zhang, P. Xu, R. Wang, Y. Tong, Q. Lu and F. Gao, *Nanoscale* 2018,
10, 13702-13712.
